# Association of Antibiotic Resistance, Cell Adherence, and Biofilm Production with the Endemicity of Nosocomial* Klebsiella pneumoniae*

**DOI:** 10.1155/2018/7012958

**Published:** 2018-09-23

**Authors:** María Dolores Alcántar-Curiel, Carmen Alejandra Ledezma-Escalante, Ma Dolores Jarillo-Quijada, Catalina Gayosso-Vázquez, Rayo Morfín-Otero, Eduardo Rodríguez-Noriega, María Lilia Cedillo-Ramírez, José Ignacio Santos-Preciado, Jorge A. Girón

**Affiliations:** ^1^Laboratorio de Infectología, Microbiología e Inmunología Clínicas, Unidad de Investigación en Medicina Experimental, Facultad de Medicina, Universidad Nacional Autónoma de México, Mexico City, Mexico; ^2^Departamento de Microbiología, Escuela Nacional de Ciencias Biológicas, Instituto Politécnico Nacional, Mexico City, Mexico; ^3^Departamento de Enfermedades Infecciosas, Hospital Civil de Guadalajara, Jalisco, Mexico; ^4^Centro de Detección Biomolecular, Benemérita Universidad Autónoma de Puebla, Puebla, Mexico; ^5^Department of Pediatrics, University of Virginia, Charlottesville, Virginia, USA

## Abstract

*Klebsiella pneumoniae *is a leading cause of multiple nosocomial infections, some of which are associated with high mortality. The increasing prevalence of antibiotic-resistant strains highlights their clinical importance and how complicated managing treatment can be. In this study, we investigated antimicrobial resistance, cell adherence, and biofilm production of nosocomial* K. pneumoniae *strains isolated from surveillance studies in a Mexican tertiary hospital and evaluated the potential association of these phenotypes with endemicity. The great majority of the clones exhibited adhesion to cultured epithelial cells and were strong biofilm producers. A direct relationship between adhesion phenotypes, biofilm production, and endemicity was not always apparent. Biofilm formation and production of ESBL did not appear to be directly associated. Notably, all the endemic strains were multidrug-resistant. This study emphasizes that while endemic strains possess various virulence-associated properties, antimicrobial resistance appears to be a determining factor of their endemicity.

## 1. Introduction

*Klebsiella pneumoniae, *a Gram-negative opportunistic pathogen, ranks among the most important causes of nosocomial infections in developing countries and among the eight most important causes in developed countries [1], especially in immunocompromised patients and those with indwelling medical devices on which this microorganism is able to grow in a biofilm. The main reservoir for transmission of* K. pneumoniae *is the gastrointestinal tract of patients and the hands of medical staff [2]. Nosocomial infections caused by* K. pneumoniae* resistant to multiple antibiotics have become an increasing public health concern. Several studies have reported hospital outbreaks due to isolates of* K. pneumoniae* resistant to third-generation cephalosporins, aminoglycosides, and quinolones [3]. The production of extended-spectrum beta-lactamases and other mechanisms of antibiotics resistance are favored by the constant horizontal transfer of antimicrobial resistance genes through mobile elements such as plasmids and transposons, which are essential factors for* K. pneumoniae *to survive in nosocomial environments [4].

Nosocomial infections are related to the elevated use of invasive techniques for diagnostic, as well as to invasive and therapeutic interventions, particularly when medical devices are introduced in different anatomical regions of patients. Microbiological studies show that bacteria, including* K. pneumoniae* grow attached on the surfaces of such devices and infected tissues, producing biofilms, in which bacteria persist for long periods of time in spite of antimicrobial therapy and the presence of an immune system. Bacterial biofilms also pose a risk for dissemination between patients and throughout the hospital [5].

An essential step in the colonization of tissues and development of infection is the adherence of bacteria to host cells through surface-associated fimbrial and nonfimbrial adhesins [6, 7]. Fimbriae are extracellular fibrillar adhesive appendages comprised of thousands of protein subunits.* K. pneumoniae *produces different virulence factors involved in pathogenesis, including capsular polysaccharide, lipopolysaccharide, fimbriae, siderophores, and serum resistance [4].* K. pneumoniae* strains express at least three different fimbrial types: mannose-sensitive type 1 fimbriae, mannose-resistant type 3 fimbriae, and the* E. coli *common pilus (ECP) [8–10]. All of these fimbriae are involved in the adherence of clinical* K. pneumoniae* isolates to host cells and in biofilm formation [9, 10].

While it appears that antibiotic resistance coupled to biofilm formation and extracellular fibrillar adhesive appendages have contributed to the global spread of* K. pneumoniae* and other gram-negative bacilli, the relationship among these factors has not been elucidated [11]. Recently, the relationship between virulence, multidrug resistance, and biofilm formation in clinical strains of* Acinetobacter baumannii* and* K. pneumoniae* was evaluated [11]. It was found that the ability to form biofilms was not associated with clonal types or multidrug resistance.

In this paper, we sought to investigate the virulence-associated properties including antibiotic resistance, cell adherence, biofilm production, and endemicity of a collection of nosocomial* K. pneumoniae *strains isolated in a Mexican tertiary hospital [12]. Our data highlights the importance and need to further investigate the bacterial and host factors that contribute to the development of nosocomial infections and the prevalence of* K. pneumoniae *isolates in hospitalized patients, some of which are responsible for recurrent outbreaks.

## 2. Materials and Methods

### 2.1. Bacterial Isolates

A collection of 168* K. pneumoniae* strains isolated from patients who developed nosocomial infections while hospitalized in the Hospital Civil de Guadalajara during a 45-month period, from February 1999 to October 2002, was evaluated for adherence to tissue cultured cells, biofilm formation on plastic, antibiotic resistance profile, and endemicity. The strains correspond to a bank of nosocomial isolates, which are part of our laboratory bacterial collection.* K. pneumoniae* ATCC700603 was employed as a positive control for type 1 pili and type 3 pili production and biofilm formation, for cell adherence this strain was used as internal control, because its level of adherence is low.* K. pneumoniae* Kpn1 and* Escherichia coli* K-12 (HB101) were used as negative controls.

### 2.2. Antimicrobial Susceptibility

The minimum inhibitory concentrations (MICs) for amikacin, gentamicin, ampicillin, aztreonam, cefepime, cefotaxime, cefoxitine, ceftazidime, cefuroxime, ticarcillin-clavulanate, tobramycin, imipenem, ciprofloxacin, levofloxacin, ofloxacin, and tetracycline were determined using the Sensititre ARIS® 2X System (TREK Diagnostic Systems Inc., Westlake, Ohio, USA) and were confirmed using an agar dilution method, according to Clinical Laboratory Standards Institute (CLSI) guidelines [13]. In all multidrug-resistant strains, production of extended spectrum beta-lactamases (ESBLs), including ceftazidime and cefotaxime, was screened with or without clavulanate by the double disk synergism method [13].* K. pneumoniae *ATCC 700603 and* E. coli* ATCC 25922 were used as positive and negative controls, respectively.

### 2.3. Genotyping by Pulsed-Field Gel Electrophoresis (PFGE)

To determine the genetic relatedness of isolates, PFGE analysis was performed as previously described [14, 15]. Chromosomal DNA of* K. pneumoniae* isolates was prepared as described elsewhere [15]. DNA fragments were prepared by* Xba*I (New England Biolabs, Beverly, MA) restriction analysis and separated by PFGE using a GenePath System (Bio-Rad®). Restriction fragment analysis was used to define clonal related or unrelated isolates according to the Tenover criteria [16].

### 2.4. PCR Detection of Fimbrial Genes

The presence of type 1 pili, type 3 pili, and ECP (*fimA, mrkA, *and* ecpA*) major structural genes was determined by DNA amplification.* fimA *was amplified using primers fimA-F 5'CGG ACG GTA CGC TGT ATT TT-3' and* fimA*-R, 3'-GCT TCG GCG TTG TCT TTA TC-5',* mrkA* was amplified using primers G593, 5'CGG TAA AGT TAC CGA CGT ATC TTG TAC TG-3' and G594 3'GCT GTT AAC CAC ACC GGT GGT AAC 5', and* ecpA* was amplified using primers G569, 5'GCA ACA GCC AAA AAA GAC ACC-3' and G570, 3'-CCA GGT CGC GTC GAA CTG-5', as previously described [10]. A subset of amplified products was nucleotide sequenced and compared to pilin nucleotide sequences on the database GenBank. Additionally, strains producing type 1 pili were detected by the yeast-agglutination assay. Briefly, 20 *μ*L of a 24-h bacterial culture in Dulbecco's minimal essential medium (DMEM) containing high concentration of glucose was mixed with 20 *μ*L of a* Saccharomyces cerevisiae* suspension on a glass slide [17]. The presence of type 3 pili on the bacteria was evidenced by slide agglutination of tannic acid treated human erythrocytes (3%) using 100 *μ*L of overnight bacterial cultures obtained in LB medium and DMEM high glucose [18].

### 2.5. Cell Adherence

The adherence assays were performed in quadruplicate employing human cervical HeLa cells seeded on 24-well polystyrene plates [18]. Three wells served to quantitate adhering bacteria and one well to visualize the adherence phenotypes by Giemsa staining and light microscopy. Briefly, cell monolayers at 80% confluence were infected with 10^7^ bacteria of overnight LB medium or DMEM cultures and incubated for 3 h at 37°C in a humidified atmosphere with 5% CO_2_. The wells were washed two times with PBS (pH 7.4), treated with 0.1% Triton X-100, ten-fold serially diluted, and plated onto LB agar plates to quantitate the attached bacteria as colony-forming-units (CFUs). These experiments were performed in triplicate on 3 different days and the mean values were expressed as adhering CFUs. The data obtained were statistically analyzed using One-way-Anova and Tukey multiple comparison test and GraphPad Prism 7.04. A replica sample was fixed with 300 *μ*L of methanol, stained with Giemsa, and mounted on glass slides. The stained samples were visualized with a Nikon Eclipse microscope T 300-E.

### 2.6. Biofilm Production

Bacteria were grown statically in DMEM at 37°C during 3 h and adjusted to 1x10^10^ CFU/mL. The biofilm assay was performed in triplicate in 24-well polystyrene plates containing 400 *μ*L of DMEM with high glucose per well. One hundred *μ*L of the bacteria was added to each well and incubated statically for 24 h at 37°C. After washing 3 X with PBS the biofilms formed were fixed with 1 mL of 2% formaldehyde for 20 min, washed, and then stained with 0.5% Crystal Violet for 20 min. The plates were washed exhaustively with distilled water and 1 mL of 95% ethanol was added to recover the absorbed stain. The dye was read in a spectrophotometer at 595 nm [10] and the values obtained were expressed as a biofilm production index (BPI).* K. pneumoniae* ATCC 700603 was used as a positive control with an assigned BPI value of 1.0 while* E. coli* K12 (HB101) was used as the negative control (BPI =0).

## 3. Results

### 3.1. Antimicrobial Susceptibility

Among the 168* K. pneumoniae* isolates studied, 121 (72.0%) were resistant to ceftazidime, 115 (68.5%) to cefotaxime, 163 (97%) to cefuroxime, 137 (81.5%) to aztreonam, 125 (74.4%) to gentamicin, and 113 (67.2%) to amikacin. The MIC_50_ to cefotaxime and ceftazidime in these strains was >128 *μ*g/mL (Table 1). All ceftazidime and cefotaxime resistant isolates were positive for ESBL production. All isolates were susceptible to imipenem and 6% were resistant to ciprofloxacin.

### 3.2. Genotyping and Epidemiological Analysis

**Figure 1:**
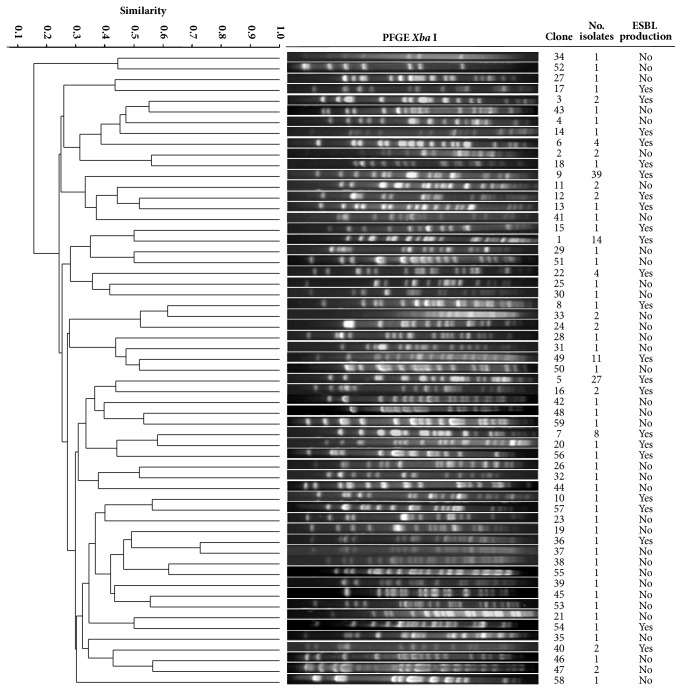
Dendrogram representing genetic relationships among 168* K. pneumoniae* strains. One representative strain of each PFGE pattern (clone) identified is presented. ESBL-producing* K. pneumoniae* were part of 23 different PFGE patterns.

**Figure 2:**
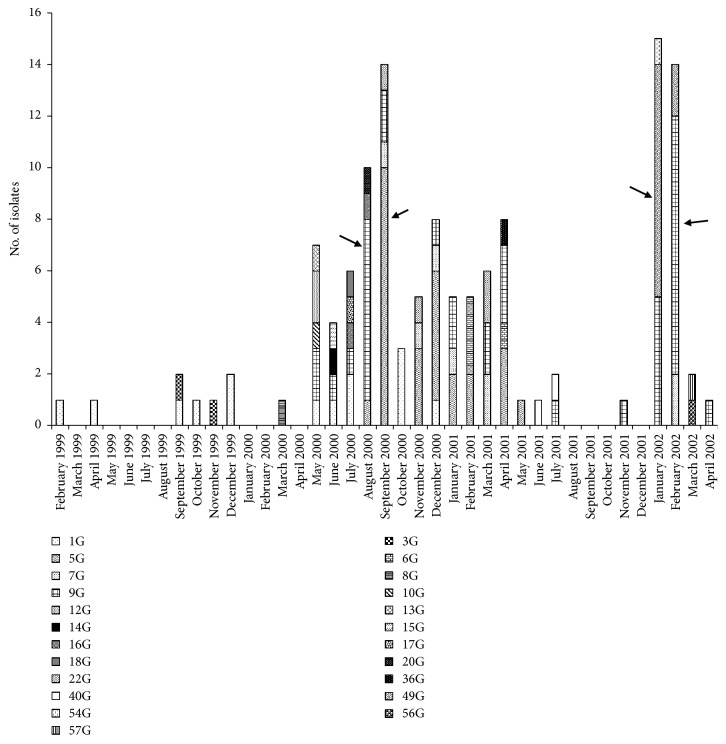
ESBL-producing* K. pneumoniae *clones identified during a 45-month surveillance study period. A total of 23 clones were detected in 127 ESBL-producing* K. pneumoniae* strains. Arrows show four different outbreaks of bacteremia caused by* K. pneumoniae*.

Among the 168 isolates, we found 59 different clones of which 23 of them (containing 127 isolates) were ESBL producers (Figure 1). Ceftazidime-resistant (CAZ^R^) clones G5 (27 isolates) and G9 (39 isolates) were responsible for 40% of the total nosocomial infections. The Department of Hospital Epidemiology at the Hospital Civil de Guadalajara reported 4 outbreaks of bacteremia due to* K. pneumoniae* during 2000-2002 (Figure 2). The first outbreak included 7 cases of bacteremia due to clone G9 in the Neonatal Intensive Care Unit 1 (NICU1) in August 2000. The second outbreak took place shortly thereafter in September 2000 in NICU2 involving 10 bacteremia cases due to clone G5. Isolates of both clones were detected for the first time in both NICU wards since the beginning of year 2000 but had been endemic for almost 8 months before they produced outbreaks. After these outbreaks, clones G5 and G9 were isolated in these wards and reported as endemic. A year and a half later (January/February 2002), clone G9 caused another outbreak involving 15 bacteremia cases in NICU2. At the same time a fourth outbreak occurred in NICU1 and in the General Pediatric ward, involving 10 bacteremia cases caused by a newly introduced clone G49. After this last outbreak and until the end of the study in October 2002 no nosocomial infections due* Kpn*CAZ^R^ isolates were reported. In all, 25% of the 168 nosocomial infection cases caused by* K. pneumoniae* isolates were outbreak strains and 75% were considered endemic.

### 3.3. Detection of fimA, mrkA, and ecpA Genes

All of the 168 isolates tested agglutinated yeast cells indicating they all produce type 1 pili. However, only 78% (131) were positive for the presence of* fimA *gene. This result is not surprising since the* fimA* gene is subject to antigenic and phase variation leading to On and Off states of piliation. Although 100% of the studied isolates carried the* mrkA* gene, only 66% (111) of them produced type pili 3 as determined by phenotypic tests. Regarding the frequency of the ECP, 91% (153/168) of the isolates contained the* ecpA* gene. These data indicate that 3 fimbrial types are highly conserved among the* Klebsiellae* and that they must be important for the biology of these organisms.

### 3.4. Cell Adherence

**Figure 3:**
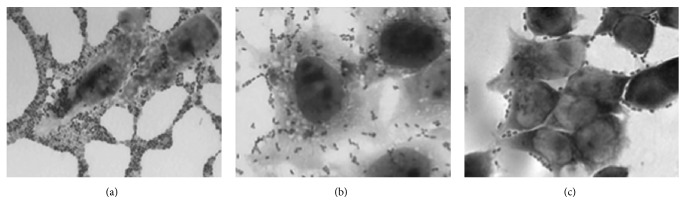
HeLa cell adherence phenotypes exhibited by* K. pneumoniae *strains. The collection of* K. pneumoniae *strains was tested for adherence to HeLa cells and we identified 3 levels of adherence: high, moderate, and poor. These micrographs were obtained after staining of the infected monolayers with Giemsa solution and visualize using light microscopy. Images were taken at 63X. (a)* K. pneumoniae* 232/01 (clone 47) showing high level of cell adherence with bacterial aggregates between neighboring cells. (b)* K. pneumoniae* 821 (clone 9) adheres moderately to the cells and (c)* K. pneumoniae *373 (clone 4) adheres poorly.

To evaluate the adhesiveness of the* K. pneumoniae *strains to HeLa cell monolayers, a representative isolate of each of the 59 clones was randomly selected. Based on the level of adherence the strains were classified as follows: highly adherent (>1x10^7^ CFU/well), moderately adherent (1-9x10^6^ CFU/well), and poorly adherent (<1x10^5^ CFU/well). Notably, 100% of these clones were PCR positive for type 3 pili genes while the presence of ECP and type 1 pili genes varied between 80-91% and 60-70%, respectively. We found that only 5/59 (8.5%) of the clones were highly adherent (Table 2, Figure 3) while the great majority of the clones 48/59 (81.4%) exhibited moderate adhesion to HeLa cells (Table 2, Figure 3). The remaining 6 clones (10.2%) were poorly adherent (Table 2, Figure 3). In sum, 91% of the clones studied were moderately to highly adherent to HeLa cells and possessed the 3 fimbrial genes to different extent. Because clones 5 and 9 were the most prevalent, we analyzed whether there was a significant difference in adherence between the strains of the same clone. Three representative strains of each of these two clones were selected for this analysis. Isolates 591, 097/01, and 588 of clone 5 and isolates 277/01, 373, and 821 of clone 9 exhibited moderate adherence, suggesting that this phenotype is an important attribute of these strains.

### 3.5. Biofilm Production

Sixty-nine percent (41/59) of the clones were strong biofilm producers and showed a BPI >1, 20.3% (12/59) were weak biofilm producers (BPI <1), and 10.1% (6/59) did not produce biofilm (BPI =0) (Table 2).

### 3.6. Correlation between Adherence, Biofilm Production, Antimicrobial Resistance, and Endemicity of K. pneumoniae Clones

Most of the highly adherent clones had a BPI >1 while the vast majority (35 clones) of the moderately adherent clones had BPI >1, 9 clones had a BPI <1, and 4 clones were nonbiofilm producers (BPI =0). Among the poorly adherent clones 3, 2, and 1 had BPIs >1, <1 or 0, respectively (Table 2).

The most frequent clones with the greatest periods of endemicity were clones 1, 5, 7, 9, and 49 (Figure 2). Clone 9, which had the greatest period of endemicity (24 months), contained the largest number of isolates (Figure 2, Table 3). Among isolates of clone 9, the great majority (61.5%) were weak biofilm producers (BPI <1), 10.2% were strong biofilm producers (BPI >1), and a significant percentage of the isolates (28.2%) were nonbiofilm producers (BPI =0) (Table 3). Interestingly, all of the isolates belonging to clone 9 were resistant to ceftazidime.

Clone 5, the second most frequent clone, had a 10-month period of endemicity (Figure 2, Table 3) and showed a high level of adherence to HeLa cells. The first group of isolates of clone 5 obtained during the first four months showed a BPI <1, while the second group of isolates obtained over the next five months had a BPI >1 (Table 3). All isolates belonging to this clone were resistant to ceftazidime.

Clones 1, 7, and 49 persisted for 23, 18, and 10 months in the hospital environment, respectively (Figure 2, Table 3). Interestingly, although these clones exhibited moderate cell adhesion, they showed a BPI >1 and were all resistant to ceftazidime (Table 3).

Clones 11, 24, and 47 showed a significant increase in BPI from the first to the second isolation. In addition, one isolate from clone 47 was sensitive to ceftazidime, while the second isolate was ceftazidime-resistant. In contrast, the first isolate of clone 22 was a strong biofilm producer (BPI >1) but the next consecutive four isolates had a BPI <1. The remaining 50 clones were comprised of 1 or 2 isolates, of which 59% showed high biofilm production and, of these, 77% were sensitive to ceftazidime (Table 2).

## 4. Discussion

In the last decade,* K. pneumoniae *has emerged among the 5 most important pathogens causing nosocomial infections in Mexico [12], and as one of the most prevalent ESBL-producing isolates in a tertiary care teaching hospital in Guadalajara, Jalisco, Mexico [12]. This motivated us to study antimicrobial resistance, clonality, endemicity, and other virulence properties of the strains responsible for several outbreaks in this setting. Our study strongly suggests that antimicrobial resistance plays a crucial role in the persistence of* K. pneumoniae *in the hospital environment. This notion is based on the finding that a significant percentage 39% of the clones (23/59) produced ESBL, of which clones 5 and 9 caused major outbreaks in two years, and were designated as endemic clones. These data are consistent with the outcome of other recent studies in which* K. pneumoniae* and other ESBL-producing enterobacterial strains from cases of nosocomial infections were reported [12, 19–21].

In addition to antimicrobial resistance, cell adhesion and biofilm formation are other attributes that have been extensively studied and have been linked to the virulence and persistence of* K. pneumoniae* in the hospital environment. Adherence is the first step in host colonization and in the formation of biofilms. The adherence of* K. pneumoniae* is mediated by multiple fimbrial types [10]. We studied the relationship between the ability of these isolates to adhere to HeLa cells and the production of type 1, type 3 pili, and ECP. We found that all isolates had the* mrkA* type 3 pili gene and the majority of strains (~78%) contained* fimA*, regardless of their level of cell adherence. ECP was identified in 91% and 80% of the isolates showing moderate and high levels of adherence, respectively. Type 3 pili and ECP appears tightly associated with the adhesion properties of* K. pneumoniae,* a finding consistent with previous studies [10, 22]. These data indicate that these 3 fimbrial types are highly conserved among nosocomial* K. pneumoniae* and that they must be important for the biology of these organisms and in their interaction with their human host.

Since there is an association between cell adherence and biofilm development, we expected that the manifestation of these phenotypes was directly related among the 168 isolates tested however, this was not the case. This can be explained by the fact that cell adherence and biofilm formation on non-biological surfaces can be mediated by similar or different bacterial adherence factors, induced by host cell factors not present on abiotic surfaces, and by the intrinsic nature of epithelial cells compared to glass or plastic surfaces.

Another observation from this study is that the formation of biofilms and production of ESBL are not directly associated. For example, endemic clones 1, 5, 7, and 49 produced ESBL and formed high levels of biofilm. In contrast, endemic clone 9 clone was ESBL producer but was only a moderate biofilm producer. Most interesting and intriguing was to find that nonendemic clones produced strong biofilms independent of ESBL production. In agreement with our results are the recent observations by de Campos et al., [11] who did not find a link between antimicrobial resistance and biofilm production in clinical isolates of* K. pneumoniae* and* A. baumannii*. However, our results contrast with other previous studies in which a direct relationship between the antimicrobial resistance and biofilm production has been shown [23–25]. These authors base their argument on the fact that under antibiotic pressure, mostly with a subinhibitory concentration of antimicrobials such as cefotaxime, biofilm formation was enhanced [25]. Alternatively, others workers have proposed that some plasmid-encoded resistance genes such as* ampR,* [26], modulate fimbrial gene expression, and consequently biofilm formation.

Among all the isolates belonging to each of the five clones with the highest endemicity rates in this study, multidrug resistance was the factor which was always intrinsically associated among them. However, while cellular adhesion and the ability to form biofilms may contribute to the persistence of these pathogens in patients and in the environment, these factors were not always detected in* K. pneumoniae *endemic isolates.

Finally, our data underscores the importance of conserving nosocomial isolates in preserved culture collections so that as new technologies are developed we can further investigate the bacterial and host factors that contribute to the natural history of* K. pneumoniae *nosocomial infections, their prevalence in hospitalized patients, and in particular those responsible for recurrent outbreaks.

## 5. Conclusions

Currently,* K. pneumoniae* has reached high levels of endemicity in hospitals and is one of the main causes of nosocomial outbreaks. The results on our study show that the nosocomial* K. pneumoniae* strains analyzed were found to be heterogeneous in nature and their endemicity can be largely attributed to their multidrug-resistance. This study emphasizes that while endemic strains possess various virulence-associated properties, antimicrobial resistance appears to be the determining factor of their endemicity.

## Figures and Tables

**Table 1:** Antibiotic resistance phenotype of the 168 *K. pneumoniae *nosocomial isolates.

Antibiotic	% Resistance (*n*)	MIC_50_ (*μ*g/ml)	MIC_90_ (*μ*g/ml)
AMK	67.2% (113)	64	>128
AMP	100 % (168)	32	>128
ATM	81.5% (137)	>128	>128
FEP	8.9% (15)	8.0	16
CTX	68.5% (115)	>128	>128
FOX	4.1% (7)	8.0	16
CAZ	72.0% (121)	>128	>128
CXM	97.0% (163)	>8.0	>8.0
CIP	6.5% (11)	0.125	1.0
GEN	74.4% (125)	>128	>128
IPM	0% (0)	1.0	2.0
LVX	7.1% (12)	0.5	2.0
OFX	3.5% (6)	0.25	1.0
TET	83.9% (141)	64	64
TOB	54.1% (91)	64	64
TIM	36.3% (61)	16/2	>256/2

AMK, amikacin; AMP, ampicillin; ATM, aztreonam; FEP, cefepime; CTX, cefotaxime; FOX, cefoxitin; CAZ, ceftazidime; CXM, cefuroxime; CIP, ciprofloxacin; GEN, gentamicin; IPM, imipenem; LVX, levofloxacin; OFX, ofloxacin; TET, tetracycline; TIM, ticarcillin-clavulanic acid; TOB, tobramycin.

**Table 2:** Adherence phenotype, biofilm formation, multidrug resistance phenotype and endemicity of 59 nosocomial clones of* K. pneumoniae* strains.

Clone/number of isolates	Adherence phenotype (*n*)	BPI (*n*)	MDR phenotype (*n*)	Endemicity (*n*)
G2/2, G11/2, G39/1, G44/1, G45/1	High (5)	>1 (4)	Negative (5)	Non endemic (5)
		<1 (1)		

G1/14, G3/2, G5/27, G6/4, G7/8, G8/1, G9/39/, G10/1, G12/2, G13/1, G14/1, G16/2, G17/1, G18/1, G19/1, G20/1, G21/1, G23/1, G24/2, G25/1, G26/1, G28/1, G30/1, G31/1, G32/1, G33/2, G34/1, G35/1, G36/1, G37/1, G38/1, G40/2, G41/1, G42/1, G43/1, G46/1, G48/1, G49/1, G50/1, G51/1, G52/1, G53/1, G54/1, G55/1, G56/1, G57/1, G58/1, G59/1	Moderate (48)	>1 (35) <1 (9) 0 (4)	Positive (23) Negative (25)	Endemic (6) Non endemic (42)

G4/1, G22/4, G47/2, G27/1, G29/1, G15/1	Poor (6)	>1 (3)	Positive (5)	Non endemic (6)
		<1 (2)	Negative (1)	
		0 (1)		

BPI = Biofilm production index. MDR = multidrug resistance. See text for description of adherence phenotypes.

**Table 3:** * K. pneumoniae *belonging to the five most frequent and endemic clones and phenotype of biofilm production.

Isolate number	Isolation date	Clone	Biofim Production Index (OD)	Isolate number	Isolation date	Clone	Biofim Production index (OD)	Isolate number	Isolation date	Clone	Biofim Production index (OD)
316	02/24/1999	G1	2.0	794	01/08/2001	G5	1.4	908	03/09/2001	G9	0.1
319	04/28/1999	G1	1.7	796	01/09/2001	G5	1.6	931	04/05/2001	G9	0.1
323	09/14/1999	G1	1.7	823	02/10/2001	G5	1.5	932	04/09/2001	G9	0.1
325	10/27/1999	G1	2.1	828	02/16/2001	G5	1.3	96/01	04/09/2001	G9	0.1
328	12/15/1999	G1	1.7	100/01	04/04/2001	G5	1.5	120/01	07/18/2001	G9	0.2
329	12/15/1999	G1	1.7	99/01	04/04/2001	G5	1.4	293/01	11/22/2000	G9	0.1
365	05/19/2000	G1	1.5	97/01	04/10/2001	G5	0.2	262/01	01/03/2002	G9	0.2
471	06/29/2000	G1	1.6	107/01	05/10/2001	G5	1.5	261/01	01/14/2002	G9	0.6
469	07/14/2000	G1	1.4	601	09/25/2000	G7	1.4	265/01	01/17/2002	G9	0.1
472	07/17/2000	G1	1.3	816	11/26/2000	G7	3.5	266/01	01/17/2002	G9	2.1
604	10/03/2000	G1	2.0	818	12/28/2000	G7	1.4	277/01	01/30/2002	G9	0.3
605	10/04/2000	G1	2.1	899	01/17/2001	G7	3.5	281/01	02/02/2002	G9	0.7
606	10/10/2000	G1	1.2	826	03/01/2001	G7	3.8	282/01	02/04/2002	G9	0.3
817	12/08/2000	G1	1.8	902	02/02/2001	G7	3.0	288/01	02/06/2002	G9	0.1
588	08/22/2000	G5	1.1	369/01	02/11/2002	G7	0.9	289/01	02/06/2002	G9	0.2
591	09/10/2000	G5	0.9	393/01	02/11/2002	G7	1.6	364/01	02/06/2002	G9	0.3
592	09/12/2000	G5	1.1	373	05/03/2000	G9	1.4	292/01	02/10/2002	G9	0.1
593	09/12/2000	G5	0.7	372	05/06/2000	G9	0	366/01	02/11/2002	G9	0
594	09/14/2000	G5	0.8	466	06/28/2000	G9	0	370/01	02/12/2002	G9	0
596	09/22/2000	G5	0.6	467	07/14/2000	G9	0	365/01	02/12/2002	G9	2.0
597	09/22/2000	G5	0.7	522	08/02/2000	G9	0.2	392/01	02/12/2002	G9	0
598	09/22/2000	G5	0.8	516	08/03/2000	G9	0.3	400/01	04/02/2002	G9	0.3
599	09/22/2000	G5	0.5	517	08/04/2000	G9	0.3	267/01	01/23/2002	G49	2.4
835	09/22/2000	G5	0.3	519	08/06/2000	G9	0	272/01	01/25/2002	G49	2.1
602	09/25/2000	G5	0.5	586	08/97/2000	G9	0	276/01	01/25/2002	G49	0.9
815	11/13/2000	G5	0.6	523	08/10/2000	G9	1.0	358/01	01/26/2002	G49	2.2
814	11/14/2000	G5	0.7	526	08/25/2000	G9	0	279/01	01/30/2002	G49	1.3
607	11/20/2000	G5	0.6	600	09/22/2000	G9	0.1	280/01	01/30/2002	G49	1.4
788	12/13/2000	G5	1.7	603	09/28/2000	G9	0.2	360/01	01/30/2002	G49	2.4
789	12/23/2000	G5	1.8	813	12/06/2000	G9	0	361/01	01/31/2002	G49	2.1
790	12/25/2000	G5	2.0	821	01/18/2001	G9	0.2	284/01	02/01/2002	G49	1.0
791	12/25/2000	G5	2.2	901	01/24/2001	G9	0	285/01	02/01/2002	G49	1.5
793	12/25/2000	G5	1.6	906	03/02/2001	G9	0.2	283/01	10/01/2002	G49	0.8

BPI>1 = strong biofilm producer. BPI<1 = weak biofilm producer. BPI = 0 non-biofilm producer.

## Data Availability

The data used to support the findings of this study are included within the article.
